# Transient transformation of *Podosphaera xanthii* by electroporation of conidia

**DOI:** 10.1186/s12866-014-0338-8

**Published:** 2015-02-06

**Authors:** David Vela-Corcía, Diego Romero, Juan Antonio Torés, Antonio De Vicente, Alejandro Pérez-García

**Affiliations:** Instituto de Hortofruticultura Subtropical y Mediterránea “La Mayora”, Universidad de Málaga - Consejo Superior de Investigaciones Científicas (IHSM-UMA-CSIC), Departamento de Microbiología, Universidad de Málaga, Bulevar Louis Pasteur 31 (Campus Universitario de Teatinos), 29071 Málaga, Spain; Instituto de Hortofruticultura Subtropical y Mediterránea “La Mayora”, Universidad de Málaga - Consejo Superior de Investigaciones Científicas (IHSM-UMA-CSIC), Estación Experimental “La Mayora”, 29750 Algarrobo-Costa, Málaga, Spain

**Keywords:** Electrotransformation, Hygromycin B resistance, MBC resistance, Powdery mildew fungi, β-tubulin

## Abstract

**Background:**

Powdery mildew diseases are a major phytosanitary issue causing important yield and economic losses in agronomic, horticultural and ornamental crops. Powdery mildew fungi are obligate biotrophic parasites unable to grow on culture media, a fact that has significantly limited their genetic manipulation. In this work, we report a protocol based on the electroporation of fungal conidia, for the transient transformation of *Podosphaera fusca* (synonym *Podosphaera xanthii*), the main causal agent of cucurbit powdery mildew.

**Results:**

To introduce DNA into *P. xanthii* conidia, we applied two square-wave pulses of 1.7 kV for 1 ms with an interval of 5 s. We tested these conditions with several plasmids bearing as selective markers hygromycin B resistance (*hph*), carbendazim resistance (*TUB2*) or GFP (*gfp*) under control of endogenous regulatory elements from *Aspergillus nidulans*, *Neurospora crassa* or *P. xanthii* to drive their expression. An *in planta* selection procedure using the MBC fungicide carbendazim permitted the propagation of transformants onto zucchini cotyledons and avoided the phytotoxicity associated with hygromycin B.

**Conclusion:**

This is the first report on the transformation of *P. xanthii* and the transformation of powdery mildew fungi using electroporation. Although the transformants are transient, this represents a feasible method for the genetic manipulation of this important group of plant pathogens.

## Background

Obligate biotrophic fungi are important plant pathogens that cause enormous losses in food and forage crops [1]. In contrast to necrotrophic parasites, which kill their hosts quickly, obligate biotrophic parasites establish a long-lasting interaction with their host plants [2] and are strictly dependent on a living host to complete their life cycle [3]. Powdery mildews (*Erysiphales*) are among the most common and recognisable of all plant diseases and are responsible for greater losses, in terms of crop yield, than any other single type of plant disease [4]. Important crops, including cereals, grapevine and a number of vegetables and ornamentals, are among their major targets [4]. Powdery mildew diseases remain among the most important plant pathological problems worldwide; in crop protection, the largest use of fungicides is for the control of powdery mildews [5].

Numerous vegetable crops are susceptible to powdery mildew, but cucurbits are arguably the group that is most severely affected [6]. In Spain, as in many other countries around the world, *Podosphaera xanthii* (synonym *Podosphaera fusca*) is considered to be the main causal agent of powdery mildew in cucurbits and is one of the most important limiting factors in cucurbit production [7,8]. However, despite the economic importance of this fungus, very little is known about the physiological and molecular processes involved in the biology and pathogenesis of *P. xanthii* [6]. Its nature as an obligate biotrophic parasite has imposed severe limitations to experimentation in *P. xanthii* as in the rest of powdery mildew fungi. Fortunately, these limitations are currently being overcome by the use of novel physiological, genetic and molecular techniques [9-12].

Elucidating the molecular basis of the intricate interaction between obligate biotrophs and their host plants has proven difficult because of three factors: (i) the impossibility of producing an authentic host-parasite interface *in vitro*, (ii) the lack of a method to stably transform obligate biotrophs, and (iii) the obligate selection of transformants on the host plant. In this sense, reliance on certain fungicides as selective agents that only harm the fungus but not the host could be a straight-forward approach in cases where the allele conferring the corresponding resistance is known [13]. Most publications describing the transient transformation of biotrophic fungi involve delivery of the transgenes using particle bombardment [14-16]; however, variations in helium pressure or target distance greatly affect transformation success [14,15]. Regarding powdery mildew fungi, the only two reports describing transformation experiments are those by Christiansen and co-workers [17] and Chaure and co-workers [18]. However, following these pioneer works, this methodology was not developed further due to the lack of reproducibility.

Electroporation has been successfully used to transform a number of fungi [19-21]. Electroporation is a molecular biology technique in which a high-voltage electric pulse is applied to create a population of small, aqueous pores in the cell membrane through which large molecules (e.g., DNA) either diffusely or electrophoretically enter the cell [19]; however, the manner in which DNA passes through the fungal cell wall is still unclear. While most reports involve the electroporation of protoplasts, a few researchers have used mycelia or conidia [22-25]. The use of conidia is not only simpler but also more reliable than protoplast transformation and provides more uniform results because rapidly dividing cells, such as conidia, are more receptive to plasmid integration than non-dividing protoplasts [26]. Electroporators often have multiple electrical wave form pulse settings such as exponential decay, time constant and square wave. Square wave electroporation is characterized by the voltage delivered, the duration of the pulse, the number of pulses and the length of the interval between pulses. This type of electroporation pulse allows the delivery of a more defined and regulated electrical pulse and has been associated with high transformation efficiencies in a variety of systems [27], including fungi [28].

In our laboratory, 454 sequencing of *P. xanthii* transcriptome is currently underway, which should provide considerable information about genomic data for the first time in this fungus. In this scenario, a method for functional genomics analysis of *P. xanthii* genes is needed. Therefore, the aim of this study was to explore the possibilities of genetically transforming *P. xanthii* by the electroporation of conidia. We showed that by electrotransformation, it is possible to introduce genetic material in *P. xanthii* conidia, although this transformation proved to be transient. Despite the transient transformation phenomenon observed, our results are encouraging enough to continue this line of research.

## Methods

### Plant and fungal material and culture conditions

The *P. xanthii* isolate 2086 was grown on zucchini (*Cucurbita pepo*) cotyledons cv. Negro Belleza (Semillas Fitó), a cultivar very susceptible to powdery mildew, and maintained *in vitro* as previously described [29]. Plants were grown from seeds in a growth chamber at 25°C under a 16 h photoperiod. When needed, the antifungals hygromycin B and carbendazim were used at 100 and 600 μg ml^−1^, respectively. Fungicides were sprayed onto zucchini cotyledons until runoff. Cotyledons were left to dry before deposition of conidia [30].

*Aspergillus nidulans* strain A4 was routinely grown on PDA plates and incubated at 37°C. When needed, carbendazim was used at 2 μg ml^−1^ [31].

### Construction of plasmids

The plasmids used in fungal transformation are listed in Table 1. The plasmid pCPXBteGFP containing the *egfp* gene, which encodes an enhanced variant allele of GFP, and the *TUB2* gene, a selective marker that confers resistance to methyl-2-benzimidazole carbamate (MBC) fungicides such as carbendazim or thiophanate-methyl, was constructed using the backbone of plasmid pCPXHY1eGFP [32]. The *P. xanthii TUB*2 gene (accession no. KC333362), including its promoter and terminator regions, was amplified from the MBC-resistant *P. xanthii* isolate SF60 [30] using the pair of primers Btale-F and Btale-R (Table 2), appending *Apa*I restriction sites at the 5′ and 3′ ends, respectively. The fragment was subsequently cloned into the *Apa*I-digested pCPXHY1eGFP, generating the plasmid pCPXBteGFP. The plasmid was first introduced in *A. nidulans* to evaluate the selective markers prior to transforming *P. xanthii*.

**Table 1:** **Plasmids used for** ***P. xanthii*** **electroporation and their main features**

**Plasmid designation**	**Size (bp)**	**Resistance and/or reporter genes**	**Reference**
pAN7-1	6756	*E. coli* hygromycin B phosphotransferase gene (*hph*), under the control of *A. nidulans gpd* (glyceraldehyde-3-phosphate dehydrogenase) promoter	[33]
pGPDGFP	6400	Green fluorescent protein gene (*gfp*) fused to the *gpd* promoter and the tryptophan synthetase (*trpC*) terminator from *A. nidulans*	[34]
pIGPAPA	5910	*E. coli hph* under the control of *A. nidulans trpC* promoter/*gfp* under the control of *IsL* (isocitrate lyase) promoter from *N. crassa*	[35]
pCPXHY1eGFP	9100	*E.coli hph* under the control of *A. nidulans trpC* promoter/Enhanced green fluorescent protein gene (*egfp*) under the control of *A. nidulans gpd* promoter	[32]
pCPXBteGFP	11630	Carbendazim resistant β-tubulin gene (*TUB*2) from *P. xanthii* under the control of its own promoter/*egfp* under the control of *A. nidulans gpd* promoter	This study

**Table 2:** **Primers used in this study**

**Target gene**	**Primer name**	**Sequence (5′- 3′)**	**Product size (bp)**
*TUB2*	Btale-F	GGGCCCGCCTCAGCCTATGCCATTCG	4000
	Btale-R	GGGCCCAGTCAAGACCCGGAGCA	
*hph*	hph-F	GACATCACCATGCCTGAACT	1700
	hph-R	GGTAACGCCAGGGTTTTCCC	
*egfp*	GFP-F	CTGAAGTTCATCTGCACCACC	600
	GFP-R	CTTTACTTGTACAGCTCGTCC	

### Electroporation of fungi

For electroporation of the *A. nidulans* A4 strain, conidia were used to inoculate 400 ml of PDB broth at a density of 1 × 10^7^ conidia ml^−1^, which was incubated at 37°C in a rotary shaker (300 rpm) for 7 h. Conidia were harvested by centrifugation and resuspended in 400 ml of ice-cold sterile water, centrifuged again, resuspended in 25 ml of ice-cold pre-treating YED buffer (1% yeast extract, 1% glucose in 20 mM HEPES buffer pH 8.0) and incubated for 1 h at 30°C in a rotary shaker at 100 rpm. After incubation, conidia were centrifuged and resuspended in 2.5 ml of ice-cold electroporation buffer (10 mM Tris–HCl pH 7.5, 270 mM sucrose, 1 mM lithium acetate), adjusted to 10^9^ conidia ml^−1^ and kept on ice. For electroporation, 10 μg of plasmid DNA was added to 150 μl of ice-cold conidial suspension. The final volume was adjusted to 200 μl with sterile distilled water; the mixture was incubated on ice for 15 min and then transferred to a 0.2-cm electroporation cuvette. Electrotransformation was performed using the Gene Pulser Xcell Electroporation System (Bio-Rad). Voltage was adjusted to 1.0 kV and capacitance to 25 μF; resistance was 400 Ohms. Following electroporation, 1 ml of ice-cold YED was added to the cuvette and the conidial suspension was transferred to a sterile 10 ml tube, kept on ice for 15 min and incubated at 30°C for 90 min in a rotary shaker at 100 rpm. Conidia subjected to electroporation were propagated on PDA plates supplemented with carbendazim (2 μg ml^−1^) at 37°C.

For electroporation of *P. xanthii*, a conidiospore suspension adjusted to 10^6^ conidia ml^−1^ was made in 5 ml of electroporation buffer (1 mM HEPES pH 7.0, 50 mM mannitol, 0.01% Tween-20). A volume of 120 μl of cell suspension was placed in pre-chilled 0.2-cm electroporation cuvettes and kept on ice. The conidial suspension was gently mixed with 10 μg of plasmid and subjected to an electric pulse using a Gene Pulser Xcell Electroporation System. Transformation was conducted by application of a square-wave electroporation pulse [28] as follows: two pulses of 1 ms duration at 1.70 kV with an interval of 5 s. Following electroporation, conidia were directly resuspended in 1 ml of cold 0.5 M mannitol and placed on ice for 10 min. Conidial suspension was sprayed on disinfected zucchini cotyledons and incubated as described previously [29] without selection pressure. For selection of transformant with hygromycin B (HygB), 7-day-old growing colonies were transferred to cotyledons treated with HygB (100 μg ml^−1^) and maintained as described above. After selection, putative transformants were transferred to cotyledons treated with a lower concentration of HygB (80 μg ml^−1^) to reduce the selection pressure, thus facilitating the growth of those colonies. For carbendazim selection, the fungicide (600 μg ml^−1^) was spread onto cotyledons 72 h after inoculation with electroporated conidia.

### Molecular analysis of transformants

Genomic DNA from potential *P. xanthii* transformants was extracted from small amounts of mycelium and subsequently amplified using the “multiple displacement amplification” (MDA) method [11]. For PCR analysis, fragments of the *hph* and *gfp* marker genes were amplified with the primer pairs hph-F/hph-R and gfp-F/gfp-R, respectively (Table 2). PCR amplifications were conducted using a proofreading Pwo SuperYield DNA Polymerase (Roche Diagnostics). The amplification conditions consisted of an initial denaturing step at 95°C for 2 min, followed by 30 cycles of 94°C for 1 min, 52°C for 1 min, 72°C for 2 min, and a final elongation step at 72°C for 2 min.

For Southern blot analysis, 10 μg of total DNA of the PCR-positive transformants were digested with *EcoR*V (Roche Diagnostics), size-fractionated on 0.8% agarose gels and blotted on positively charged nylon membranes (Whatman) [36]. Preparation of DIG-labelled probes using a fragment of the *hph* gene, hybridisation (50°C) and chemiluminescent detection were performed according to the manufacturer’s instructions (Roche Diagnostics).

### Microscopic analysis of transformants

To monitor fluorescence associated with GFP, *P. xanthii* colonies growing onto cotyledons were inspected in a confocal laser-scanning microscope (Leica) equipped with a TCS SP2 Laser (Ar/Kr, Gre/Ne, He/Ne; traditional phase contrast). GFP fluorescence was excited with a 488 nm laser line and detected at 515–530 nm. The analysis of putative transformants of *A. nidulans* for GFP fluorescence was conducted in an epifluorescence stereomicroscope AZ-100 (Nikon) with 485 and 530 nm filters.

### Immunoblot analysis

The expression of GFP protein in the potential *P. xanthii* transformants was evaluated by immunoblot analysis. A section of cotyledon-containing *P. xanthii* colonies was cut out and resuspended in 1 ml of phosphate-buffered saline and Tween-20 0.1% (Sigma-Aldrich). To break down fungal structures, sonication was employed at 80% amplitude and 3 cycles of 1 min using a UP100H sonicator (Hielscher). After sonication, sodium deoxycholate (Sigma-Aldrich) was added at 0.02% to lyse the cells and solubilise cellular and membrane components and then incubated at room temperature for 15 min. Samples were TCA (trichloroacetic acid) precipitated, and the pellet was washed twice in cold acetone. After the complete evaporation of acetone, the residue was resuspended in 1 × SDS loading buffer, electrophoresed in a 12% SDS-PAGE gel and transferred onto a PVDF membrane using the Trans-Blot Turbo electrophoretic transfer cell (Bio-Rad) at 25 V for 30 min. Blot was probed with 1:1000 anti-GFP rabbit monoclonal antibody (Rockland). A secondary anti-rabbit antibody conjugated to horseradish peroxidase (Bio-Rad) was used at a dilution of 1:20000. The blot was developed using the Pierce super signal detection system following the supplier’s recommended procedures (Pierce).

## Results

### Hygromycin B resistance is not suitable for *in planta* selection

Hygromycin B resistance is one of the genetic markers most commonly used in the transformation of filamentous fungi. Therefore, we initially tried to introduce into *P. xanthii* different plasmids containing the hygromycin B resistance cassette using electroporation (Table 1). To test the sensitivity of *P. xanthii* to hygromycin B, the isolate 2086 was inoculated onto zucchini cotyledons treated with different concentrations of hygromycin B (10–1000 μg ml^−1^) to determine the minimal inhibitory concentration (MIC). The MIC value determined for isolate 2086 was 100 μg ml^−1^, and thus, this was the concentration used for selection of *P. xanthii* transformants after transformation of the conidia.

**Figure 1:**
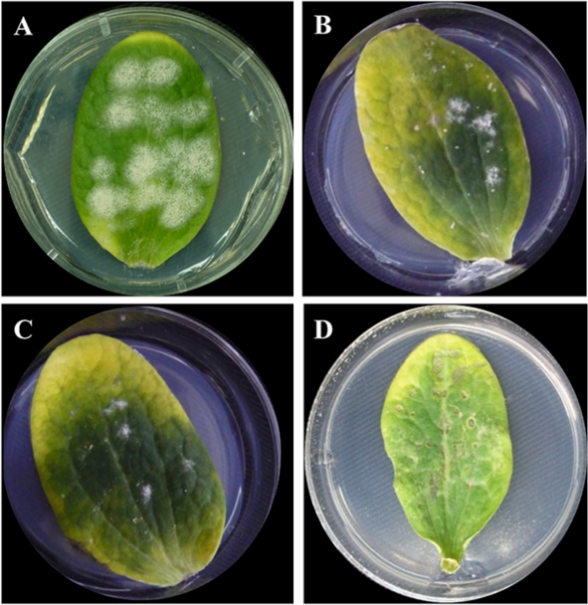
**Transformation of** ***P. xanthii*** **conidia with the plasmid pAN7-1. (A)** Untransformed conidia grown normally on non-treated zucchini cotyledons. **(B-C)** Transformants of *P. xanthii* grown on zucchini cotyledons treated with hygromycin B after electroporation of conidia with the plasmid pAN7-1. Note the reduced size of the colonies compared with untransformed colonies. No colonies were observed on cotyledons treated with hygromycin B and inoculated with untransformed conidia. **(D)** In many cases, treatment with hygromycin B induced phytotoxicity (necrotic spots) on cotyledons. Pictures were taken 15 d after electroporation of conidia.

Initially, 10 transformation experiments were performed with the plasmids containing the hygromycin B resistance cassette (*hph*) and the plasmid pGPDGFP (*gfp* reporter gene). Putative *P. xanthii* clones resistant to hygromycin B were obtained only with the plasmids pAN7-1 and pCPXHY1eGFP and only in some experiments. No transformants were obtained with the plasmids pIGPAPA or pGPDGFP. In all cases, no hygromycin B-resistant colonies were observed on control cotyledons inoculated with non-electroporated conidia. After transformation with pAN7-1, only a few colonies (0–3 colonies per cotyledon) of putative transformants resistant to hygromycin B were obtained, which were clearly visible 15 days after electroporation (Figure 1B-C). However, these colonies hardly grew compared with the positive controls, that is, conidia electroporated but not treated with hygromycin B (Figure 1A). No hygromycin B-resistant colonies were observed on negative controls of conidia electroporated in the absence of plasmid DNA. In addition, hygromycin B induced serious damage to cotyledon tissue in many cases, severely reducing its lifetime and negatively affecting the further growth of potential transformants (Figure 1D).

To verify molecularly the putative hygromycin B resistant transformants, the hygromycin B resistance cassette (*hph*) borne in plasmid pAN7-1 was amplified by PCR. Only three out of eight selected colonies yielded the expected product of 1.7 Kb, and no amplification signal was obtained on DNA from untransformed colonies (data not shown). Since a positive PCR result does not necessarily means the integration of the construct into the fungal genome, the three positive transformants were further assessed by Southern blot using a fragment of the *hph* gene as a probe. The three independent transformants gave an identical hybridisation pattern and no hybridisation signal was observed on DNA of untransformed colonies (data not shown). Since integration in the same place is so unlikely, especially when there is no sequence for targeted homologous recombination in the plasmid, it is much likely that the hybridizing bands observed were due to the presence of the plasmid in fungal cells rather than the integration of the construct into the fungal genome.

**Figure 2:**
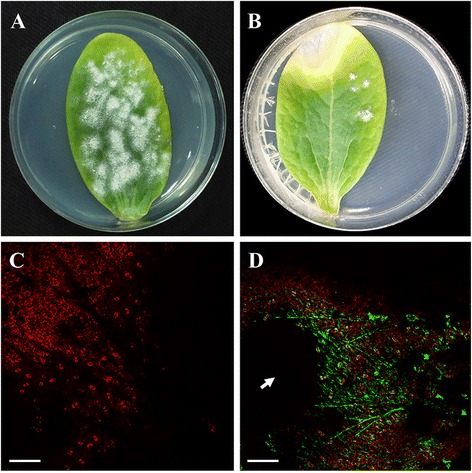
**Transformation of** ***P. xanthii*** **conidia with plasmid pCPXHY1eGFP. (A)** Untransformed colonies grown normally on non-treated zucchini cotyledons. **(B)** Transformants of *P. xanthii* obtained with pCPXHY1eGFP grown onto hygromycin B treated cotyledons. Note the reduced colony size and the damage on the host tissues caused by hygromycin B. **(C-D)** CLSM analysis of *P. xanthii* transformants. **(C)** An untransformed colony with no GFP fluorescence signal. **(D)** A transformant colony showing GFP fluorescence in the peripheral hyphae, whereas conidia did not show fluorescence (arrow). Pictures were taken 15 d after electroporation of conidia. Scale bars 100 μm.

**Figure 3:**
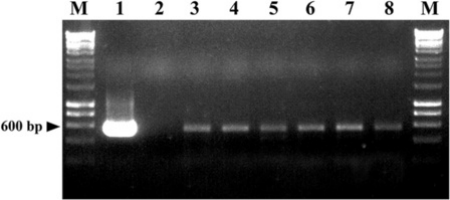
**Molecular analysis of** ***P. xanthii*** **transformants obtained after electro-transformation with plasmid pCPXHY1eGFP.** Genomic DNA from potential transformants was subjected to PCR amplification of the *egfp* gene using the primer pair GFP-F and GFP-R. The size of the expected PCR product is indicated on the left. Lanes are: 1, the plasmid pCPXHY1eGFP; 2, DNA from an untransformed colony; 3–8, potential hygromycin B-resistant and fluorescent transformants. M, molecular size marker 1 kb ladder.

The plasmid pAN7-1 does not harbour an additional reporter gene. We thus decided to include in our analysis the plasmid pCPXHY1eGFP that harbours the *hph* gene as a selection marker and also the enhanced green fluorescence protein gene (*egfp*), permitting the dual selection of transformants based on fungicide resistance and colony fluorescence. As previously observed for the plasmid pAN7-1, only a few transformants resistant to hygromycin B (0–4 colonies per cotyledon) were obtained that showed the same growth limitations (Figure 2B), whereas untransformed colonies did not show any growth defect (Figure 2A). Transformants obtained with pCPXHY1eGFP were screened for eGFP fluorescence using confocal laser scanning microscopy (CLSM) after 15 days of growth on hygromycin B-treated cotyledons. All the colonies resistant to hygromycin B were also fluorescent. An EGFP fluorescence signal was located within the hyphae and was uniformly distributed (Figure 2D), but it was not observed over the mass of conidiospores (arrow). No signal was detected in control hyphae from untransformed colonies (Figure 2C). In this case, the plant material underwent a rapid degradation, which together with the slow growth of transformant colonies hampered subsequent molecular analysis. Nevertheless, PCR analysis of the transformants could be undertaken using the primer pair GFP-F/GFP-R (Table 2), which targets the corresponding *egfp* gene. Amplification yielded the expected product of 600 bp in the six transformants analysed (Figure 3), suggesting the presence of the construct in the fungal cells. These transformants survive only two rounds of cultivation in presence of hygromycin B and, therefore, Southern blot analysis could not be performed to verify integration. However, the instability of phenotype suggests that there was no integration.

### Development of a selectable genetic marker based on the *PfTUB2* gene and MBC resistance

**Figure 4:**
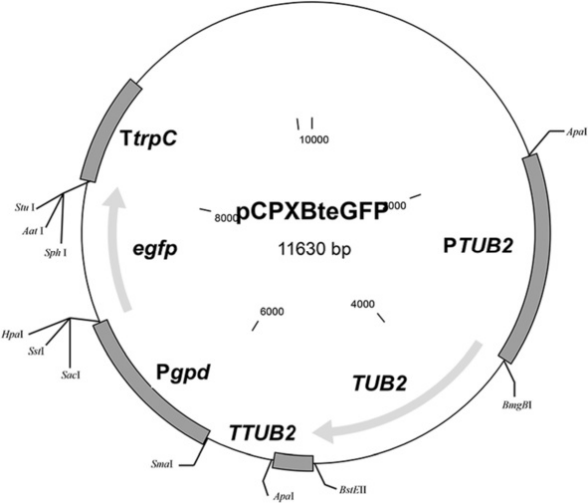
**Schematic representation of the plasmid pCPXBteGFP.** The plasmid (11.63 Kb) is a derivative of plasmid pCPXHY1eGFP that contains the *egfp* gene under the control of the Pgpd promoter and the TtrpC terminator of the *A. nidulans gpd* and *trpC* genes, respectively. The vector also contains the complete *TUB*2 gene of *P. xanthii*, which confers resistance to MBC fungicides such as carbendazim. Positions of the main restriction enzyme sites are indicated.

**Figure 5:**
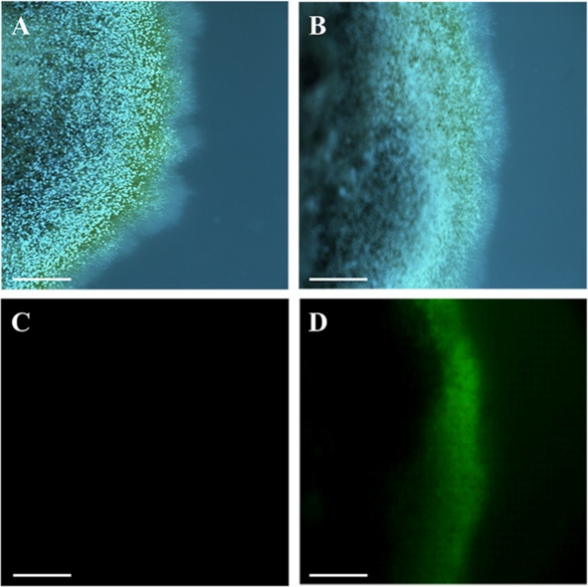
**Transformation of** ***A. nidulans*** **A4 with the plasmid pCPXBteGFP. (A-B)** Bright-field images of an untransformed colony **(A)** and a potential transformant growing in the presence of carbendazim **(B)**. **(C-D)** Epifluorescence stereomicroscopic analysis of a nontransformant colony showing no fluorescence **(C)** and a potential *A. nidulans* transformant showing fluorescence in the peripheral hyphae **(D)**. Scale bars 2 mm.

Our findings in the transformation experiments described above indicated that hygromycin B was not suitable for the selection of *P. xanthii* transformants because the phytotoxicity to zucchini cotyledons compromised normal fungal growth (Figure 1D). Therefore, we focused our efforts on the design of a new transformation vector that carried a different drug resistance cassette. For this purpose, we constructed the plasmid pCPXBteGFP (Figure 4), which contains a complete copy of the *PfTUB2* gene, including its own promoter and terminator sequences. This allele of *P. xanthii TUB2* encodes for a β-tubulin insensitive to MBC fungicides, such as carbendazim, characterised by the presence of the typical E198A substitution conferring resistance to these fungicides [30]. In addition, this plasmid carries the reporter gene *egfp* (Figure 4). The functionality of this plasmid was initially tested on the fungal strain *A. nidulans* A4, which is more amenable genetically than *P. xanthii*. After electrotransformation with pCPXBteGFP, spores of *A. nidulans* were plated onto PDA medium supplemented with carbendazim (2 μg ml^−1^). Transformants resistant to carbendazim became visible 4 days after incubation and were screened for EGFP fluorescence using an epifluorescence stereomicroscope. All the colonies showed fluorescence signals at the borderline of the colony but not over the mass of spores at the centre of the colony (Figure 5). No background fluorescence was observed in untransformed *Aspergillus* colonies.

**Figure 6:**
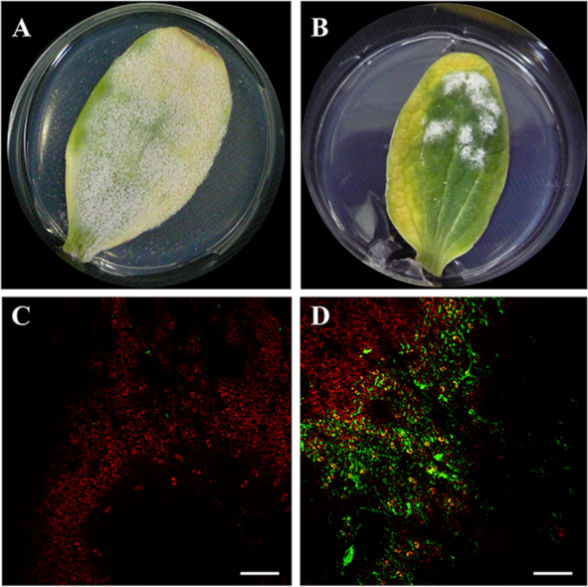
**Transformation of** ***P. xanthii*** **conidia with the plasmid pCPXBteGFP. (A)** Untransformed colonies growing normally on non-treated cotyledons. **(B)** Growth of *P. xanthii* transformants resistant to carbendazim. Conversely to hygromycin B, almost no appreciable tissue damage was observed on zucchini cotyledons. **(C-D)** EGFP analysis of transformants by CLSM analysis. **(C)** No fluorescence signal was observed in untransformed colonies. **(D)** In resistant transformants the EGFP fluorescence signal was observed within the peripheral hyphae; however, no fluorescence was observed in conidia or subjacent hyphae. No colonies were observed on cotyledons treated with carbendazim and inoculated with untransformed conidia. Pictures were taken 15 d after electroporation of conidia. Scale bars 100 μm.

Once the suitability of plasmid pCPXBteGFP was confirmed, we addressed the transformation of *P. xanthii*. As previously observed with the plasmids pAN7-1 and pCPXHY1eGFP, *P. xanthii* transformants were clearly visible 15 days after electroporated conidia were inoculated onto zucchini cotyledons treated with carbendazim (Figure 6A). These transformants were screened for EGFP fluorescence by CLSM analysis. The fluorescent signals associated with the colonies were weak and followed a distribution pattern similar to that observed with the pCPXHY1eGFP plasmid, that is, fluorescence signals distributed within and along the hyphae (Figure 6D). In addition, no fluorescence signal appeared over the mass of conidiospores (Figure 6D), which was reminiscent of the results obtained with *A. nidulans*. Although carbendazim did not provoke the severe damage to zucchini cotyledons induced by hygromycin B, potential transformants did not grow as quickly as expected.

**Figure 7:**
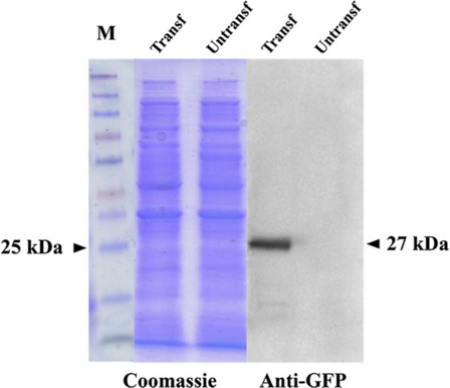
**Western blot analysis of EGFP of** ***P. xanthii*** **transformants obtained with pCPXBteGFP.** The total protein content from potential transformants and untransformed colonies was TCA-precipitated, separated by SDS-PAGE, blotted on PVDF membranes and immunoprobed with anti-GFP antibodies (1:1000). The size of the major cross-reacting band is indicated on the right. The same amount of protein (1 μg ml^−1^) was loaded per lane. M, molecular size marker Spectra Multicolor Broad Range Protein Ladder (Fermentas).

Since PCR analysis only detect the presence of the construct, these transformants were screened for the accumulation of the EGFP protein by immunoblot analysis using an anti-GFP antibody at dilution 1:1000 to verify the expression of the *egfp* reporter gene. The total protein content from potential transformants and untransformed colonies were separated in SDS-PAGE gels and blotted for the immunodetection of EGFP. A signal band corresponding to the EGFP protein (27 kDa) was observed in the protein extract of transformants that was absent in the protein extract of untransformed *P. xanthii* colonies (Figure 7).

As previously found for the plasmid pCPXHY1eGFP, the transformants obtained with the plasmid pCPXBteGFP lost resistance to carbendazim and EGFP fluorescence after two rounds of subculture and, therefore, Southern blot analysis could not be performed to verify integration. As indicated above, the instability of phenotypes demonstrated the transitory nature of this genetic transformation and suggested the absence of integration as the most obvious reason to explain the loss of phenotypes.

## Discussion

An efficient transformation system is an essential tool for genetic manipulation and functional genomics studies [37]. Different methods such as protoplast fusion, electroporation or *Agrobacterium*-mediated transformation have been widely used to transform a number of fungal species. However, the intrinsic nature of powdery mildew fungi as biotrophic parasites diminishes the possibilities of transformation. Protoplast fusion, which uses polyethylene glycol (PEG) to deliver DNA into protoplasts [38], is not applicable because obligate biotrophs are not able to regenerate from protoplasts [13]. Biolistic transformation was developed as a method for incorporating plasmid DNA into intact, thick-walled fungal cells [39]. This method involves tungsten particles coated with DNA, which are accelerated at high velocity directly into fungal spores or hyphae. Biolistic transfection has been used to transform a number of filamentous fungi such as *A. nidulans, Neurospora crassa*, *Magnaporthe grisea*, *Trichoderma harzianum* [40,41] and powdery mildew fungi. Indeed, two separate works described the successful use of biolistic transfection to transform powdery mildews [17,18], but unfortunately, no further publications have appeared reproducing the same technique, indicating that this methodology is far from being a routine approach.

Electroporation has been demonstrated as a highly efficient and fast transformation method for several fungal species [26,28,31,37,42,43]. Moreover, it has been used as an alternative means of genetic transformation for animal cells, plant protoplasts, yeast and bacteria [23,40]. Here, we report the transient transformation of the cucurbit powdery mildew fungus *P. xanthii* by electroporation of conidia using a square-wave pulse that creates transitory small pores along the membrane surface, making the cell membrane more permeable [19]. The method required high amounts of DNA (10 μg) because a high percentage of plasmid DNA is most likely damaged during the electroporation procedure [44]. By this method we could successfully introduced plasmid constructs in *P. xanthii* conidia since fungal colonies were obtained on cotyledons treated with hygromycin B or carbendazim that also showed GFP fluorescence associated with hyphae. Furthermore, the presence of the introduced constructs in the putative transformants was molecularly detected by PCR and Southern blot and even the expression of GFP protein could be detected by Western blot. However, fungal growth was slow and reduced compared with wild type and fungicide resistance and GFP expression phenotypes were lost after two rounds of cultivation of those transformants in presence of selection pressure; strongly suggesting that the constructs did not integrate into the *P. xanthii* genome.

Most of the plasmids used in our electroporation experiments carried the hygromycin B resistance gene (*hph*). This selective marker has been successfully used for a number of fungi [43,45-47]; however, it is not suitable for the selection of *P. xanthii* transformants, given the severe phytotoxicity on cotyledons. To overcome this limitation, we constructed a new vector that confers resistance to carbendazim, a fungicide belonging to the methyl benzimidazole carbamate (MBC) family [48] widely used in agriculture [49,50]. MBC resistance has been included as a selective marker in the genetic manipulation of other fungi, including powdery mildews. A mutant version of β-tubulin conferring resistance to MBC was used to construct a plasmid to transform the barley powdery mildew *Blumeria graminis* f. sp. *hordei* using a biolistic delivery system [17,18]. MBC fungicides inhibit tubulin polymerisation affecting cell division, vesicle transport, cell shape, intracellular transport, and cell motility [51]. In *P. xanthii*, resistance to MBC is due to the substitution E198A in the *PfTUB*2 gene [30]. Therefore, we opted for an MBC fungicide such as carbendazim as a selective marker because it does not exhibit phytotoxic effects, is a widely used fungicide and has been used for selection of powdery mildew transformants in the past. Unfortunately, as observed for hygromycin B resistance, a slower growth of the putative transformants was observed that could also result from selection pressure, as if native β-tubulin is still produced in the transformants this will probably interfere with carbendazim and reduce growth. In any case, carbendazim resistance also disappeared after two rounds of cultivation, again suggesting that the construct did not integrate into the genome. Additional selectable markers such as bialaphos or sulfonylurea resistance [18,52] should be used in future experiments.

Along with a drug resistance gene, additional features must be present on plasmids for fast identification of the transformants and proper visualisation of fungal structures. Fluorescent marker genes such as GFP are particularly useful tools for *in situ* monitoring of bacteria and fungi [53,54]. Unlike other biomarkers, GFP does not require any substrate or additional cofactors to fluoresce, and even single cells expressing GFP can be observed by either fluorescence or CLSM microscopy [55]. In our experiments, the transformants lost the ability to show fluorescence signal and to grow in the presence of hygromycin B or carbendazim after a few rounds of sub-cultivation. Once again, the most obvious reason for the loss of GFP fluorescence is that the plasmids did not integrate into the fungal genome.

Compared with other procedures, electroporation of conidia is, in principle, an easy and rapid method to transform powdery mildew fungi. In this work, using *P. xanthii* as a model powdery mildew species, we were able to deliver different plasmids and genetic markers within the conidia, although this method has been shown to be transient and the resulting transformants were unstable. Transient transformation has been widely used for the transformation of many non-model plant pathogenic fungi, such as the rust fungus *Melampsora lini* [56], *Rosellinia necatrix* [57], or the biotrophic fungus rust *Puccinia graminis* f. sp. *tritici* [16]. In addition, it has been used to analyse virulence-related genes such as the *AVR* genes [56]. Transient transformation is likely the best approach when a stable transformation method is not available. Therefore, this method could suitable for molecular analysis of non-model fungal plant pathogens such as the powdery mildews, among others, that are recalcitrant to stable transformation.

To obtain stable transformants, vectors with homologous sequences could be used to facilitate integration into the genome by means of homologous recombination. Neutral loci should be first identified and then, plasmids with large flanking homologous sequences could be constructed that allow direct integration by double homologous recombination events in such loci. Furthermore, in order to increase the frequency of transformation, some protocols recommend the use of lineal plasmids [58]. In our study we always used circular plasmids. In further studies, transformation with linearized versions of the constructs should be tested in order to increase the frequency of integration.

## Conclusions

The development of a reliable transformation method is the “Holy Grail” of powdery mildew research. Our results show that electrotransformation could be a route for the delivery of DNA into powdery mildew cells. Therefore, efforts must be made to pursue the stability of the transgenes. Once proper conditions for transformation and stable integration of the constructs have been achieved, a new tool for the functional analysis of powdery mildew genes would become available that would allow examining, for example, specific powdery mildew genes such as candidate effector genes and their role in pathogenesis and/or disease resistance.
